# Decrement of choroid vascularization during spontaneous migraine attacks: An optical coherence tomography angiography study

**DOI:** 10.1111/ene.16568

**Published:** 2024-11-25

**Authors:** Marina Romozzi, Vincenzo Trigila, Giovanni Cuffaro, Sofia Marcelli, Luigi Francesco Iannone, Paolo Calabresi, Gustavo Savino, Catello Vollono

**Affiliations:** ^1^ Dipartimento Universitario di Neuroscienze Università Cattolica del Sacro Cuore Rome Italy; ^2^ Neurologia, Dipartimento di neuroscienze, Organi di Senso e Torace Fondazione Policlinico Universitario Agostino Gemelli IRCCS Rome Italy; ^3^ Dipartimento di neuroscienze, Organi di Senso e Torace Università Cattolica del Sacro Cuore Rome Italy; ^4^ Oculistica Fondazione Policlinico Universitario A. Gemelli IRCCS Rome Italy; ^5^ Section of Clinical Pharmacology and Oncology, Department of Health Sciences University of Florence Florence Italy; ^6^ Neurofisiopatologia, Dipartimento di neuroscienze, Organi di Senso e Torace Fondazione Policlinico Universitario Agostino Gemelli IRCCS Rome Italy

**Keywords:** aura, FAZ, migraine, OCTA, retinal vessel density, retinopathy

## Abstract

**Objective:**

This study aimed to analyze the microcirculation of the macula, the optic nerve, and the choroid in patients with migraine by optical coherence tomography angiography (OCTA) during spontaneous migraine attacks, comparing the findings with scans performed in the interictal period in the same subjects and healthy controls (HCs).

**Methods:**

In this case‐crossover design study, patients diagnosed with migraine who underwent an OCTA during a migraine attack were enrolled. A cohort of HCs was recruited for comparison. Data from ocular and orthotic examinations and clinical and demographical information were collected. All subjects were imaged with Solix full range OCT, recording the following parameters: macular vessel density (VD), inside disc VD, peripapillary VD, disc whole image VD, fovea choriocapillaris VD, fovea VD, parafovea VD, peripapillary thickness, fovea thickness, parafovea thickness, macular full retinal thickness, and foveal avascular zone (FAZ).

**Results:**

Thirteen patients (26 eyes individually assessed) with a diagnosis of migraine were included (9 without aura [69.2%] and 4 with aura [30.7%], with a mean age of 25.2 ± 3.4 years) and scanned during the ictal and interictal phase. Fifteen age‐matched HCs (30 eyes individually assessed) were included. The foveal choriocapillaris VD was significantly lower in the ictal phase (63.3 ± 2.47%) compared to the interictal phase in the same patients (64.9 ± 2.79%) (*p* = 0.0019). Comparing the ictal scans from migraine patients and HCs, the FAZ area was significantly larger, and the inside disc, fovea, and fovea choriocapillaris VDs were significantly lower.

**Conclusion:**

The study demonstrated a dynamic decrement in choroidal vascularization in migraine patients during spontaneous migraine attacks.

## INTRODUCTION

Migraine is a common and complex neurological disease, ranking among the most disabling medical conditions, characterized by the recurrence of attacks with variable intensity and frequency [1]. Although the pathogenesis of migraine (with or without aura) is not yet totally understood, it has been proposed that there is an interaction between neural and vascular factors. The pathophysiological mechanism underlying migraine aura is cortical spreading depression (CSD), a slowly propagating wave of depolarization involving neurons and glial cells [2]. At the same time, the activation and sensitization of the trigeminovascular system, which is the major pain‐signaling pathway, leads to the release of vasodilatory neuropeptides [2].

Since neurovascular mechanisms underlie migraine pathogenesis and the cerebral and retinal microcirculation share comparable embryology and anatomy, the relation between migraine and retinal microvascular changes has been proposed [3].

Optical coherence tomography angiography (OCTA) is a new technique that has allowed non‐invasive study of retinal and choroidal vascularization in multiple diseases [4]. Only a few studies investigated retinal vessel densities with OCTA in migraine patients outside migraine attacks, showing that patients with migraine present a retinal vascular decrement [5, 6, 7]. Due to the technical difficulties of conducting exams during a migraine attack and before acute medication intake, the potential changes that occur during attacks are largely unknown [8]. Cortical spreading depression‐like phenomena, such as retinal spreading depression, which have been previously supposed to underlie retinal migraine, may also occur during a migraine attack [9]. Additionally, studies in rats showed that the retinal arterial vasculature might be modulated by neuropeptides like calcitonin gene‐related peptide (CGRP), a key molecule implicated in migraine pathogenesis and released during migraine attacks [10].

We hypothesized that there would be changes in vascular structure and function in patients with migraine between the interictal and ictal state as well as compared with healthy controls (HCs).

Herein, the microcirculation of the macula, the optic nerve, and the choroid in migraine patients, with and without aura, was analyzed by OCTA during spontaneous migraine attacks and the interictal period. Ictal and interictal OCTA findings were also compared between migraine patients with and without aura. A cohort HCs was also included for comparison.

## METHODS

### Study design and population

This is a study with a case‐crossover design conducted at the tertiary Headache Centre of the *Fondazione Policlinico Universitario Agostino Gemelli IRCCS* in Rome from 1st February 2023 to 28th February 2024. The study population included patients diagnosed with migraine with (MA) and without aura (MO). The study intervention involved performing OCTA imaging during a spontaneous migraine attack, with two control groups: the same patients themself during the interictal phase and a cohort of healthy controls. Healthy controls were volunteers recruited among staff members and faculty in the same timeframe.

Inclusion criteria for patients were: (i) age 18–60; (ii) a diagnosis of MA or MO, episodic or chronic migraine, according to the International Classification of Headache Disorders 3 (ICHD‐3) [11].

Inclusion criteria for HCs were: (i) age 18–60; (ii) no lifetime history of primary or secondary headache according to ICHD‐3; (iii) no history of psychiatric and neurological disorders.

The age at which a cut‐off was chosen was according to studies on OCTA parameters that change with aging [12].

Exclusion criteria for both patients and HCs were: (i) systemic diseases that may affect the microcirculation (including diabetes mellitus, hypertension, and rheumatological diseases); (ii) chronic intake of vasoactive drugs such as calcium channel blockers and beta‐blockers; (iii) myopic or hyperopic spherical equivalent refractive errors ≥3.00 dioptres; (iv) diagnosis of retinal migraine; (v) other neurological diseases; (vi) diseases affecting the optic nerve and the retina (including glaucoma); (vii) history of any intraocular surgery. The use of oral contraceptives was recorded due to their effects on retinal microvasculature [13, 14].

The study received approval from the Ethical Committee of the Università Cattolica del Sacro Cuore (protocol ID 4155/2021), and all patients gave written informed consent.

### Demographic and clinical data

Demographic data for both patients and HCs, including age, sex, comorbidities, and concomitant treatments, were collected by a neurologist (M.R. or C.V.) before OCTA scanning. For patients with migraine, the following data were also collected: diagnosis according to ICHD‐3; monthly migraine days (MMDs) and monthly headache days (MHDs); mean pain severity assessed with the Numerical Rating Scale (NRS); acute medication intake (type and number); migraine preventive treatments; the Migraine Disability Assessment (MIDAS) scale [15]; migraine‐related disability assessed by the Head Impact test‐6 (HIT‐6) [16].

Finally, detailed information was collected for the attack during the ictal OCTA scan: presence of aura, type of aura, nature (pulsating or not pulsating), location of pain (unilateral or bilateral), presence or absence of photophobia, presence of autonomic symptoms, NRS, timing from onset.

### Ophthalmologic examination and OCTA

A neuro‐ophthalmologist (G.C. or G.S.) performed a complete ophthalmologic examination on migraine patients and HCs, including best‐corrected visual acuity, ocular tonometry, fundoscopy, and macular and optic nerve OCTA.

For every patient, the evaluation with OCTA occurred at two distinct time points:

(i) During an untreated spontaneous migraine attack with and without aura and (ictal phase); (ii) during a headache‐free period (interictal phase), defined as the absence of migraine in the 72 h before and after the evaluation. In the interictal phase scan, a complete ophthalmological evaluation was also conducted. For HCs, the OCTA scan was performed once.

The ictal OCTA scans were not performed if: (i) the attack did not fulfill the criteria for MO or MA; (ii) the quality of the OCTA scan <8/10 (see section 2.3); (iii) intake of symptomatic medication to treat the attack before the scan; (iv) attack onset >24 h.

Eyes were examined through the Solix Fullrange OCT device (Optovue Inc., Freemont CA, USA‐version 2019 V1.0.0.305), a Spectral‐domain OCTA with high scanning and acquisition speed, able to perform 120.000 scans per second [17].

After acquiring the volumetric data sets, Motion Correction Technology (MCT), a 3D correction tool, was applied to correct image distortions, resulting in precise post‐processing outcomes. Only high‐quality scans with a signal strength of >8 were included. The same operator conducted all scans, and the physician, who was blinded to the patients' diagnoses, examined and excluded any OCTA images not meeting the quality standards or contained artifacts. The physician also manually adjusted the segmentation and propagation when the software struggled to identify retinal areas accurately. This manual adjustment was also used to delineate the FAZ area if the software failed to identify it or did so incorrectly (using 6 × 6 mm scan areas centered on the fovea).

After processing the scans, the following data on vascularization were collected: FAZ, macular vessel density (VD), disc whole image VD, inside disc VD, fovea VD, parafovea VD, peripapillary VD, and fovea choriocapillaris VD. The following structural data were collected: fovea thickness, peripapillary thickness, parafovea thickness, and macular full‐retinal thickness.

### Statistical analysis

The sample size was calculated with G*Power 3.1 software for repeated measures, based on previous studies on OCTA in migraine [18], with an effect size (Cohen's d) of 0.826, an alpha level of 0.05, and a power of 0.80, resulting in a sample size of approximately 15 patients.

Demographic characteristics were reported as mean ± standard deviation [SD] for continuous variables. Categorical variables were presented as absolute number (n) and percentage (%) and compared with the chi‐squared *χ* ^2^ test. The normality assumption was evaluated using the Kolmogorov–Smirnov test. According to the distribution of each variable, a Wilcoxon signed‐rank test or a paired *t*‐test was performed to evaluate changes in quantitative variables between the ictal and interictal phases.

Comparisons between migraine patients in the ictal and interictal phases and between patients with and without aura were conducted using either the Mann–Whitney *U* test or the *t*‐test, depending on whether the data distributions were normal. No imputation was done for missing data or missing variables; the number of patients evaluated is reported in tables and graphs.

Both eyes of patients and HCs were analyzed individually. A two‐tailed p‐value <0.05 was considered significant. Statistical data were analyzed with SPSS version 26.0 (IBM Corp. SPSS Statistics, Armonk, NY, USA).

## RESULTS

### Cohort characteristics

**FIGURE 1:**
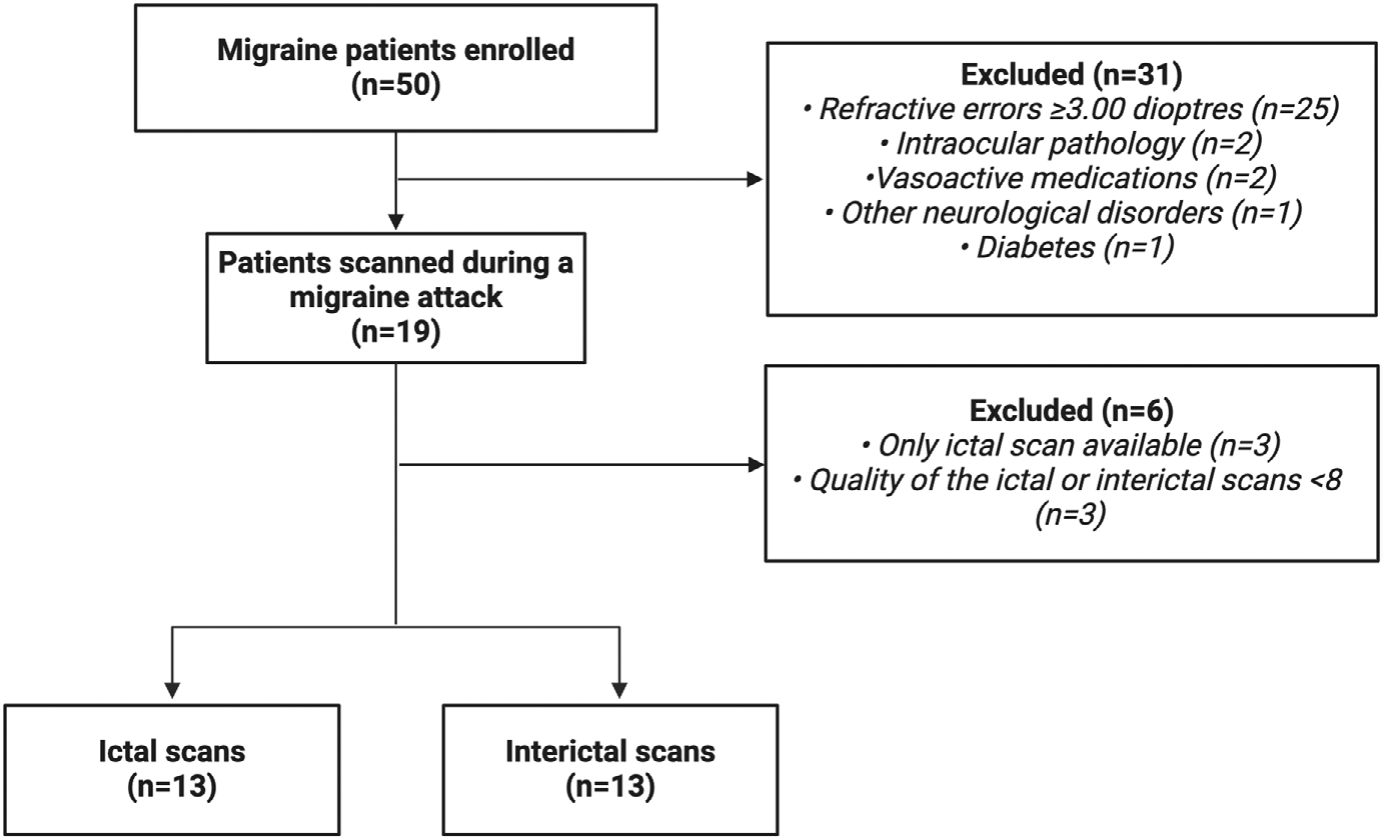
Flowchart of the study.

Thirteen patients (26 eyes individually assessed) were included with a diagnosis of migraine (9 MO [69.2%] and 4 MA [30.7%]) and a mean age of 25.2 ± 3.4 years. Fifteen age‐ and sex‐matched HCs (30 eyes individually assessed) were also enrolled, with a mean age of 27.4 ± 5.1 years. One patient was scanned during the visual aura. The study flowchart is reported in Figure 1.

Table 1 reports the clinical and demographic features of migraine patients and the characteristics of the attacks scanned.

**TABLE 1:** Clinical and demographic features of migraine patients and characteristics of the attacks scanned.

	Overall population (*n* = 13)
Demographics	
Age [years], mean ± SD	25.2 ± 3.4
Sex female, *n* (%)	10 (76.9)
Migraine features	
Disease duration (years), mean ± SD	10.4 ± 6.9
Chronic migraine, n (%)	1 (7.7)
Medication overuse, n (%)	0 (0)
Monthly migraine days, mean ± SD	4.3 ± 3.0
Monthly headache days, mean ± SD	6.0 ± 8.1
Migraine with aura, n (%)	4 (30.8)
Absolute number of analgesics, mean ± SD	4.3 ± 3.0
MIDAS score, mean ± SD	20.2 ± 16.7
HIT‐6 score, mean ± SD	60.1 ± 6.0
Attacks characteristic (ictal scan)	
Time from onset of attack (minutes)	151.5 ± 151.4
NRS score (1–10), mean ± SD	6.7 ± 2.2
Unilateral pain, n (%)	12 (92.3)
Pulsating quality of pain, n (%)	7 (53.9)
Scanned during visual aura, n (%)	1 (7.7)
Presence of photophobia, n (%)	13 (100)
Presence of autonomic features, n (%)	1 (7.7)

*Note*: Percentages are calculated on column total.

### Comparison of OCTA scans in ictal and interictal phases in the same migraine patients

**FIGURE 2:**
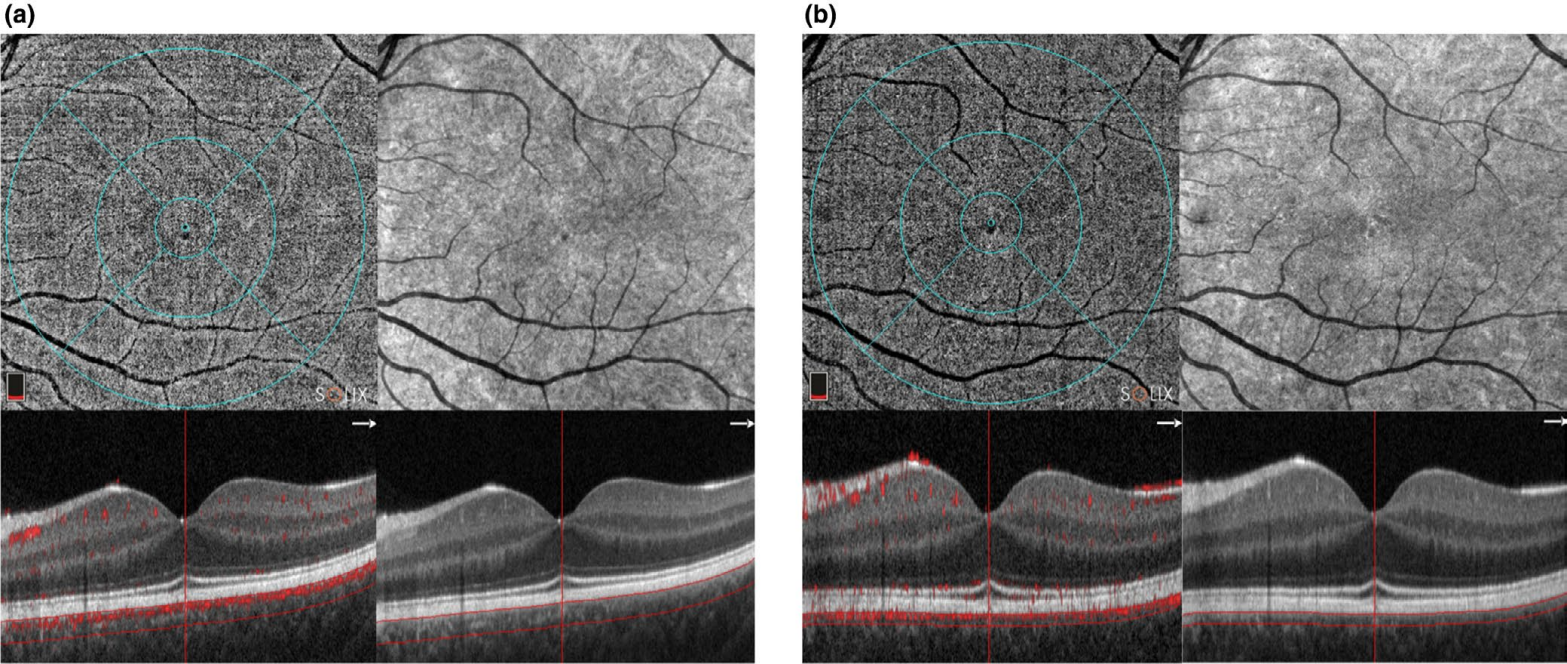
Representative macular optical coherence tomography angiography (OCTA) scans a patient with migraine during the ictal phase (a) and in the interictal phase (b). The foveal choriocapillaris vessel density (VD) decreased in the ictal phase compared to the interictal phase.

The foveal choriocapillaris VD was significantly lower in the ictal phase (63.3 ± 2.47%) compared to the interictal phase (64.9 ± 2.79%) (*p* = 0.0019) (Figure 2, Table 2). No significant difference was reported among the other OCTA parameters between the ictal and the interictal phase (Table 2).

**TABLE 2:** Optical coherence tomography angiography (OCTA) findings in migraine patients scanned during attack (ictal scan) and interictal period.

	Ictal (*n* = 26 eyes)	Interictal (n = 26 eyes)	*p*‐value^a^
FAZ (mm^2^)	0.251 ± 0.10	0.245 ± 0.11	0.206^c^
Disc whole image VD (%)	46.77 ± 2.51	46.95 ± 2.18	0.665^c^
Inside disc VD (%)	48.74 ± 4.68	50.77 ± 6.87	0.113^b^
Peripapillary VD (%)	49.72 ± 2.45	49.75 ± 2.24	0.956^c^
Peripapillary thickness (mm)	92.27 ± 8.62	92.15 ± 9.12	0.656^c^
Macular whole image VD (%)	55.66 ± 3.23	56.16 ± 2.72	0.431^b^
Fovea VD (%)	34.33 ± 7.87	36.40 ± 5.14	0.211^b^
Parafovea VD (%)	57.38 ± 3.78	58.64 ± 3.30	0.141^b^
Fovea thickness (mm)	256.11 ± 24.59	256.69 ± 25.86	0.242^c^
Parafovea thickness (mm)	322.00 ± 15.25	322.31 ± 16.43	0.563^c^
Foveal choriocapillaris VD (%)	63.34 ± 2.47	64.91 ± 2.79	**0.019** ^c^

*Note*: Values are reported as mean and standard deviation (SD). Statistically significant results (*p* < 0.05) are shown in bold type.

Abbreviations: FAZ, foveal avascular zone; HC, healthy controls; VD, vessel density.

^a^ Comparisons are calculated through parametric and non‐parametric tests for repeated measures.

^b^ Wilcoxon Signed‐Rank Test.

^c^ Paired *t*‐test.

The Comparison of ictal and interictal OCTA scans between migraine patients with and without aura did not yield significant differences.

### Comparison of OCTA scans between migraine patients (ictal and interictal phases) and healthy controls

**FIGURE 3:**
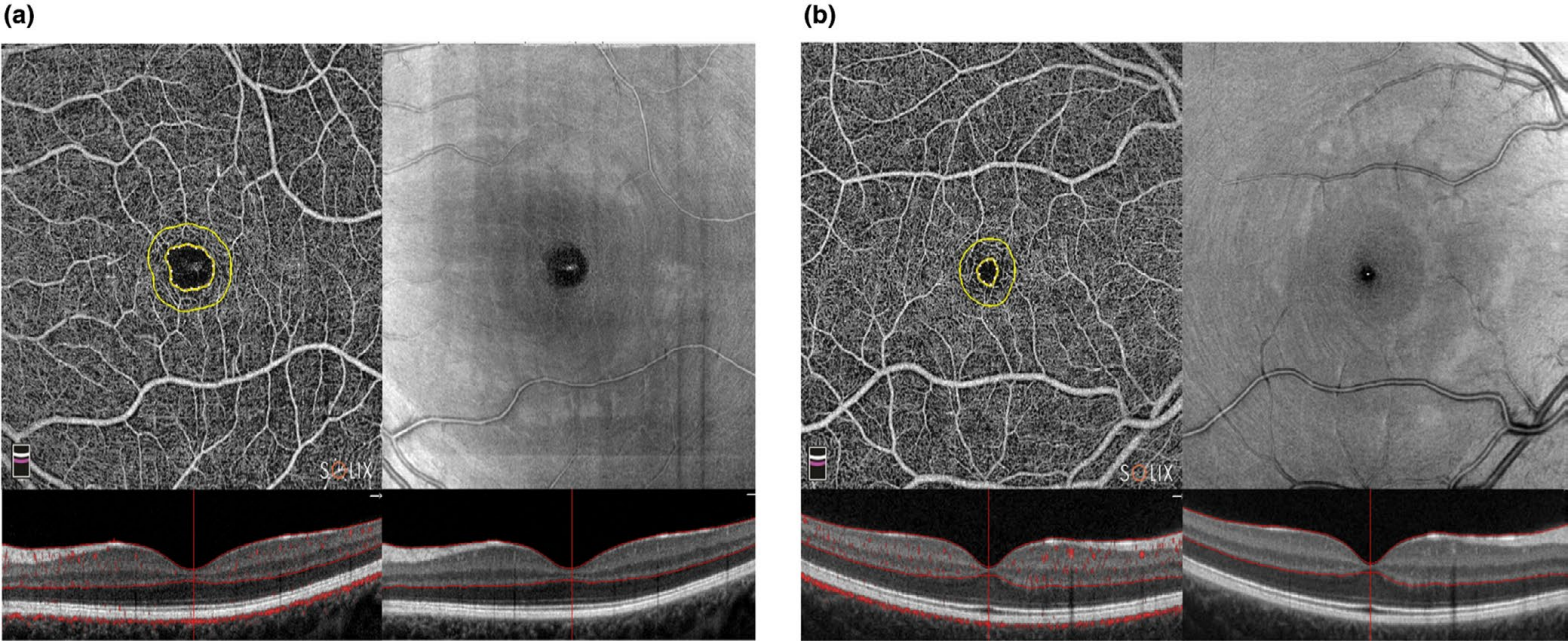
Representative macular optical coherence tomography angiography (OCTA) scans patients with migraine (a) compared with healthy controls (HCs) (b). The foveal avascular zone (FAZ) area is increased in patients with migraine compared to HCs. The FAZ area is circled in yellow.

In the comparison between the ictal phase from migraine patients and HCs, the FAZ area was significantly larger (0.251 ± 0.102 mm^2^) in the ictal phase compared to HCs (0.189 ± 0.078 mm^2^, *p* = 0.012) (Figure 3). The inside disc VD and the fovea VD were lower in patients scanned in the ictal phase compared to HCs (respectively, *p* = 0.030 and *p* = 0.035). The fovea choriocapillaris VD was significantly lower during attacks than HCs (*p* = 0.006).

Concerning the structural OCT parameters, including the foveal, parafoveal, and peripapillary thickness, there were no significant differences between patients scanned during attacks and HCs (Table 3).

**TABLE 3:** Optical coherence tomography angiography (OCTA) findings in migraine patients scanned during attack (ictal) compared to healthy controls (HCs).

	Ictal (*n* = 26 eyes)	HCs (*n* = 30 eyes)	*p*‐value^a^
Age (mean ± SD)	25.2 ± 3.4	27.4 ± 5.1	0.059
Sex, females (n, %)	10 (76.9)	7 (46.7)	0.102^b^
FAZ (mm^2^)	0.251 ± 0.10	0.185 ± 0.08	**0.010**
Disc whole image VD (%)	46.77 ± 2.51	46.7 ± 2.38	0.932
Inside disc VD (%)	48.74 ± 4.68	51.46 ± 3.70	**0.019**
Peripapillary VD (%)	49.72 ± 2.45	48.75 ± 2.52	0.150
Peripapillary thickness (mm)	92.27 ± 8.62	90.53 ± 6.75	0.402
Macular whole image VD (%)	55.66 ± 3.23	55.87 ± 2.74	0.792
Fovea VD (%)	34.33 ± 7.87	38.11 ± 5.10	**0.035**
Parafovea VD (%)	57.38 ± 3.78	58.61 ± 3.35	0.200
Fovea thickness (mm)	256.11 ± 24.59	265.77 ± 20.56	0.115
Parafovea thickness (mm)	322.00 ± 15.25	328.11 ± 14.66	0.131
Foveal choriocapillaris VD (%)	63.34 ± 2.47	64.99 ± 2.28	**0.012**

*Note*: Values are reported as mean and standard deviation (SD). Statistically significant results (*p* < 0.05) are shown in bold type.

Abbreviations: FAZ, foveal avascular zone; HC, healthy controls; VD, vessel density.

^a^ Comparisons are calculated through non‐parametric Mann–Whitney *U* test;

^b^ Comparison calculated through Chi‐squared test.

The comparison between the interictal phase of migraine patients and HC showed significant enlargement of FAZ in migraine patients (0.245 ± 0.11 mm^2^) compared to HC (0.185 ± 0.08 mm^2^) (*p* = 0.023) (Supplementary Table S1).

## DISCUSSION

In this study, patients with migraine scanned during the ictal phase showed a significant decrement in choroidal vessel densities compared to those in the interictal phase. Moreover, the comparison of ictal scans to healthy controls showed a larger FAZ area and a decrement in the choroidal, foveal, and optic disc vessel densities.

The FAZ is a round, capillary‐free area in the central macula, and enlargement of FAZ is an indirect sign of microangiopathy. Several studies have assessed the dimensions of FAZ in patients with migraine, finding a significantly larger area in migraine patients (in particular with aura) compared to healthy controls [5, 7]. On the contrary, the reduction in retinal vessel densities in patients scanned interictally compared to healthy controls yielded conflicting results [5, 7, 19].

To date, few studies have assessed choroid vascularization in patients with migraine compared to controls [20]. A study by Icoz and Colleagues found a lower luminal area, a choroidal vascular parameter, in patients with migraine with aura compared to a control group [14]. Another study reported a decrement of the foveal choriocapillaris vessel density in migraine patients with aura compared to patients without [18]. The authors hypothesized that the changes in the foveal choriocapillaris vessel density may occur at an earlier stage than the vessel density modifications in the retina [18].

Prior research has suggested that neurogenic inflammation and alterations in vascular caliber could adversely affect the vascular endothelium. Furthermore, these events could partly explain the heightened risk of vascular disorders, such as stroke and vascular retinopathy, observed predominantly in patients with migraine with aura [21].

However, very few studies assessed patients during attacks. This is due to the dynamic and unpredictable nature of migraine attacks, making it difficult to conduct these evaluations, especially before the intake of symptomatic drugs.

A report of a patient during a migraine attack with visual aura reported narrowing of the retinal vessels and decreased foveal vessel densities. These changes improved three hours after the resolution of the aura [19].

A recent study by Podraza et al. found a statistically significant difference in the vessel flux index (VFI), a composite parameter indicator of retinal perfusion, in the parafoveal region during a migraine attack compared to the interictal phase in both migraine with and without aura. However, this study failed to find significant differences considering conventional OCTA parameters (i.e., vessel densities) and did not analyze choroidal vascularization [8]. However, the study compared ictal and interictal scans of different patients.

Güler et al. conducted a study on patients scanned with OCTA during the ictal phase and found no difference in OCTA parameters, including the study of superficial and deep plexi compared to healthy controls [22]. However, the ictal scans were compared with healthy controls and not with interictal scans of the same patients. The timing of the acute attack was also not reported [22].

The findings of our study indicate a significant role of choroidal vascularization during migraine attacks. This may be attributed to the distinct characteristics of choroidal circulation compared to retinal circulation. Specifically, the choroidal circulation has a high blood flow rate, while the retinal circulation has a lower flow. Furthermore, while the retinal circulation is primarily regulated by local factors with efficient autoregulation, lacking autonomic innervation, the choroidal circulation is mainly innervated by the sympathetic nervous system and exhibits poor autoregulation [23]. This lack of efficient autoregulation in the choroidal circulation may make it susceptible to changes in blood flow and perfusion during migraine attacks, in which there is an autonomic activation [23, 24].

The finding of significant differences between the ictal and interictal phases, and in particular of a wider vascular involvement (retina and choroid) during the attack, support the hypothesis that in migraine attacks, the mechanisms already occurring during the interictal period might become more pronounced.

Furthermore, CGRP and its two receptor components (RAMP1/CRL) are present in the animal retina, but higher levels of the CGRP receptor seem to be present in the choroid than in the retina. Prieto et al. showed a significant relaxing effect of CGRP in isolated bovine retinal arteries. This observation is further reinforced by findings demonstrating that CGRP can induce significant vasodilation in the retinal arteries of rabbits in vivo [25, 26].

This study has several strengths. First, patients were rigorously selected based on strict criteria differentiating the ictal and interictal phases. Furthermore, each patient underwent comparisons within the ictal and interictal phases, assuring an intraindividual comparison of the values.

However, some limitations must be considered, particularly the small sample size and the inclusion of patients with varying degrees of migraine severity, including both episodic and chronic forms. This limitation could be attributed to the challenges associated with scanning migraine patients acutely during a migraine attack.

## CONCLUSION

Our data showed a dynamic decrement in the choroidal vascularization in migraine patients during a spontaneous attack of migraine. OCTA is a useful non‐invasive screening tool that may provide further insight into the pathogenesis of migraine, even in the ictal phase.

## AUTHOR CONTRIBUTIONS

**Marina Romozzi:** Writing – review and editing; writing – original draft; investigation; validation; visualization; methodology; conceptualization; formal analysis; project administration; data curation. **Vincenzo Trigila:** Investigation; project administration; visualization; data curation. **Giovanni Cuffaro:** Conceptualization; validation; visualization; project administration; data curation. **Sofia Marcelli:** Data curation; investigation. **Luigi Francesco Iannone:** Writing – original draft; visualization; supervision; project administration. **Paolo Calabresi:** Supervision; project administration; visualization. **Gustavo Savino:** Data curation; supervision; investigation. **Catello Vollono:** Writing – review and editing; visualization; project administration; supervision; data curation; methodology.

## FUNDING INFORMATION

This research received no specific grants from funding agencies in the public, commercial, or not‐for‐profit sectors.

## CONFLICT OF INTEREST STATEMENT

The authors declare no conflicts of interest.

## ETHICS STATEMENT

The study received approval from the Ethical Committee of the Università Cattolica del Sacro Cuore (protocol ID 4155/2021), and all patients gave written informed consent.

## Supporting information


**Table S1.** Comparison of the optical coherence tomography angiography (OCTA) findings between patients with migraine in the interictal phase and healthy controls.

## Data Availability

The data that support the findings of this study are available from the corresponding author upon reasonable request.
