# Supplementary material for: Decrement of choroid vascularization during spontaneous migraine attacks: An optical coherence tomography angiography study
Source: Eur J Neurol. 2024 Nov 25;32(1):e16568. doi: 10.1111/ene.16568 (PMC11625912; doi:10.1111/ene.16568)
Supplement: Supplementary file 1 — Table S1. Comparison of the optical coherence tomography angiography (OCTA) findings between patients with migraine in the interictal phase and healthy controls. [file ENE-32-e16568-s001.docx]

| Supplementary Table 1. Optical coherence tomography angiography (OCTA) findings in migraine patients scanned during the interictal period compared to healthy controls (HC). | | | | |
| --- | --- | --- | --- | --- |
|  | **Interictal**  **(n=26 eyes)** | | **HCs**  **(n=30 eyes)** | **p-value^a^** |
|  |  | |  |  |
| FAZ (mm^2^) | | 0.245±0.11 | 0.185±0.08 | **0.023** |
| Disc whole image VD (%) | 46.95±2.18 | | 46.7±2.38 | 0.701 |
| Inside disc VD (%) | 50.77±6.87 | | 51.46±3.70 | 0.633 |
| Peripapillary VD (%) | 49.75±2.24 | | 48.75±2.52 | 0.128 |
| Peripapillary thickness (mm) | 92.15±9.12 | | 90.53±6.75 | 0.449 |
| Macular whole image VD (%) | 56.16±2.72 | | 55.87±2.74 | 0.699 |
| Fovea VD (%) | 36.40±5.14 | | 38.11±5.10 | 0.218 |
| Parafovea VD (%) | 58.64±3.30 | | 58.61±3.35 | 0.981 |
| Fovea thickness (mm) | 256.69±25.86 | | 265.77±20.56 | 0.150 |
| Parafovea thickness (mm) | 322.31±16.43 | | 328.11±14.66 | 0.167 |
| Foveal choriocapillaris VD (%) | 64.91±2.79 | | 64.99±2.28 | 0.913 |
| *Values are reported as mean and standard deviation (SD). FAZ, foveal avascular zone; HC, healthy controls; VD, vessel density. Statistically significant results (p < 0.05) are shown in bold type. ^a^Comparisons are calculated through non-parametric Mann-Whitney U test.* | | | | |
